# Time to blood pressure control and predictors among patients receiving integrated treatment for hypertension and HIV based on an adapted WHO HEARTS implementation strategy at a large urban HIV clinic in Uganda

**DOI:** 10.1038/s41371-024-00897-3

**Published:** 2024-02-01

**Authors:** Willington Amutuhaire, Fred Collins Semitala, Isaac Derick Kimera, Christabellah Namugenyi, Frank Mulindwa, Rebecca Ssenyonjo, Rodgers Katwesigye, Frank Mugabe, Gerald Mutungi, Isaac Ssinabulya, Jeremy I. Schwartz, Anne R. Katahoire, Lewis S. Musoke, George A. Yendewa, Chris T. Longenecker, Martin Muddu

**Affiliations:** 1 Department of Medicine, Case Western Reserve University School of Medicine, Cleveland, OH USA; 2 Makerere University Joint AIDS Program (MJAP), Kampala, Uganda; 3 Makerere University Institute of Statistics and Applied Economics, Kampala, Uganda; 4 Makerere University Infectious Diseases Institute, Kampala, Uganda; 5 Department of non-communicable diseases, Uganda Ministry of Health, Kampala, Uganda; 6 Uganda Heart Institute, Kampala, Uganda; 7 Yale School of Medicine, Section of General Internal Medicine, Connecticut, CT USA; 8 Department of Medicine, Makerere University College of Health Sciences, Kampala, Uganda; 9 Department of Medicine, University Hospitals Cleveland Medical Center, Cleveland, OH USA; 10 Department of Global Health and Division of Cardiology, University of Washington, Seattle, WA USA

**Keywords:** Diseases, Hypertension

## Abstract

In this cohort study, we determined time to blood pressure (BP) control and its predictors among hypertensive PLHIV enrolled in integrated hypertension-HIV care based on the World Health Organization (WHO) HEARTS strategy at Mulago Immunosuppression Clinic in Uganda. From August 2019 to March 2020, we enrolled hypertensive PLHIV aged $\ge$18 years and initiated Amlodipine 5 mg mono-therapy for BP (140–159)/(90–99) mmHg or Amlodipine 5 mg/Valsartan 80 mg duo-therapy for BP ≥ 160/90 mmHg. Patients were followed with a treatment escalation plan until BP control, defined as BP < 140/90 mmHg. We used Cox proportional hazards models to identify predictors of time to BP control. Of 877 PLHIV enrolled (mean age 50.4 years, 62.1% female), 30% received mono-therapy and 70% received duo-therapy. In the monotherapy group, 66%, 88% and 96% attained BP control in the first, second and third months, respectively. For patients on duo-therapy, 56%, 83%, 88% and 90% achieved BP control in the first, second, third, and fourth months, respectively. In adjusted Cox proportional hazard analysis, higher systolic BP (aHR 0.995, 95% CI 0.989-0.999) and baseline ART tenofovir/lamivudine/efavirenz (aHR 0.764, 95% CI 0.637–0.917) were associated with longer time to BP control, while being on ART for >10 years was associated with a shorter time to BP control (aHR 1.456, 95% CI 1.126–1.883). The WHO HEARTS strategy was effective at achieving timely BP control among PLHIV. Additionally, monotherapy anti-hypertensive treatment for stage I hypertension is a viable option to achieve BP control and limit pill burden in resource limited HIV care settings.

## Background

Effective antiretroviral therapy (ART) has improved survival in persons living with HIV (PLHIV) [1]. This has resulted from improved programmatic HIV care world over, most importantly in sub-Saharan Africa (SSA) where the burden of disease is highest [2, 3]. With increased life expectancy, the burden of hypertension and associated cardiovascular disease in PLHIV has increased [4–6].

To address the care of hypertension in programmatic HIV settings, the Joint United Nations Program on HIV/AIDS (UNAIDS) adopted the strategy of integrating hypertension screening and treatment into already established HIV care systems [7–9]. Prospectively, multiple countries including in SSA have adopted this approach. Common challenges in these settings have been lack of basic hypertension medication, lack of training on hypertension care for frontline HIV care givers as well as limited diagnostic capabilities for secondary hypertension and complications [9–11].

The 8th Joint National Committee (JNC 8) and the European Cardiology Society guidelines for the management of hypertension recommend the initiation of hypertension treatment with preferably two agents at small doses with preference given to thiazide diuretics, calcium channel blockers and Angiotensin-converting enzyme inhibitors or Angiotensin receptor blockers in black populations. The target for treatment is optimized blood pressure control within a month, and failure of which is the recommendation to titrate medication doses and number of agents [12, 13].

To integrate hypertension care in routine HIV services at Mulago National Referral Hospital, Immunosuppression (ISS) clinic, we adapted a treatment protocol for hypertension based on the World Health Organization (WHO) HEARTS technical package for hypertension treatment [14]. The HEARTS-based treatment protocol guided clinicians on the stepwise titration of hypertension medications from either low dose monotherapy or duo-therapy depending on the patient’s baseline blood pressures [15].

In this study, we sought to determine the time to blood pressure control and its predictors among adult hypertensive PLHIV who received integrated treatment for hypertension and HIV based on the adapted WHO HEARTS protocol. Additionally, we aimed to determine if monotherapy for patients with stage I hypertension was a viable option to control blood pressure and limit pill burden in the HIV clinical setting.

## Methods

### Study setting and design

This was a prospective cohort study at the Mulago ISS clinic located within Mulago National Referral Hospital complex in Kampala, Uganda. The clinic provides comprehensive HIV services to over 16,500 PLHIV. Since August 2019, the clinic has been providing integrated HIV and hypertension care.

### Study participants and inclusion criteria

Between August 2019 and March, 2020, we enrolled consented PLHIV $\ge$18 years newly diagnosed treatment-naïve hypertensive patients while in HIV care. Patients were followed-up for 12 months up to March 2021. A diagnosis of hypertension was based on two repeat blood pressure (BP) measurements of systolic BP ≥ 140 or diastolic BP ≥ 90 mmHg or both, at least a week apart, after a five-minute rest in a sitting position in a clinic setting in accordance with the American Heart Association guidelines [16]

### Intervention

**Fig. 1:**
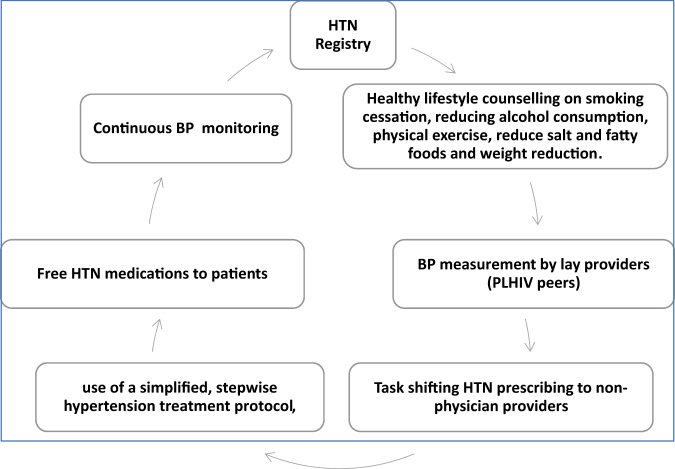
Simplified hypertension management Protocol adapted from WHO hearts model. The figure illustrates the adapted HIV- hypertension integration protocol used in the study clinic benchmarked on the WHO HEARTS model. HTN Hypertension, BP Blood pressure, PLHIV People Living with HIV.

The hypertension and HIV integration implementation package was earlier published and included, healthy lifestyle counseling, use of a simplified, stepwise hypertension treatment protocol, access to hypertension medicines to patients at no cost, prescribing of hypertension medicines by non-physician health workers and established systems for monitoring integrated care[15] (Fig. 1).

Three hypertension medicines were available for prescription by health workers: Amlodipine 5 mg and 10 mg, Valsartan- 80 mg and 160 mg and Hydrochlorothiazide- 12.5 mg and 25 mg.

**Fig. 2:**
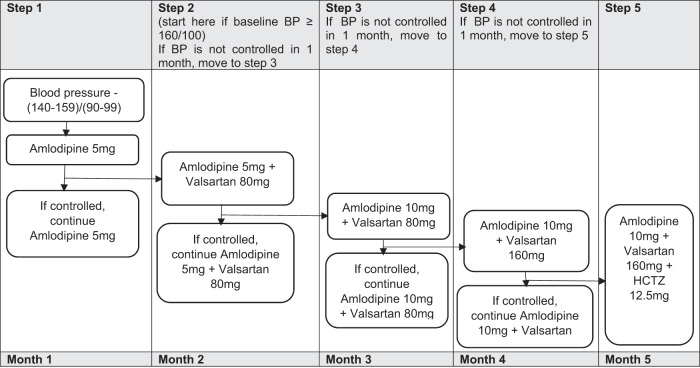
Schema for the Hypertension treatment protocol implemented at the study clinic. The figure illustrates the stepwise manner on how hypertension treatment was titrated in the study protocol. BP blood pressure, mg milligrams, HCTZ hydrochlorothiazide.

Patients with baseline BP between (140–159)/(90–99) mmHg were initiated on Amlodipine 5 mg monotherapy while those with baseline blood pressures of ≥160/90 mmHg were started on Amlodipine 5 mg and Valsartan 80 mg duo-therapy. Patients were reviewed at the clinic monthly until they attained BP control defined as a blood pressure less than 140/90 mmHg. During each clinic visit, single BP readings were taken after 5 min rest following the same baseline protocol and titration of hypertension medications was effected as shown in Fig. 2. After attaining BP control, patients received three-month refills of both hypertension and ART medications [15, 17].

The choice of medications used in the hypertension protocol was guided by the evidence on effectiveness from the CREOLE trial [18] and was further influenced by a subsidy from the medication local supplier.

### Data collection

We collected clinical, demographic and anthropometric data at baseline including sex, age, weight, height, blood pressure, current and previous ART regimens, history of diabetes mellitus (DM), smoking history, latest viral loads, baseline CD4 cell counts at the start of ART and duration on ART from hard copy patient files as primary data sources. Prospectively, blood pressure was measured during monthly reviews and later three monthly for patients who achieved both blood pressure control and HIV viral suppression.

### Statistical analyses

We described baseline characteristics using means and standard deviations for continuous variables including age and baseline CD4, and frequencies and percentages for categorical variables; sex, body mass index (BMI), duration on ART, smoking history, history of diabetes mellitus (DM) and latest viral loads. We compared baseline characteristics of patients who were started on one hypertension medicine and those that were started on two hypertension medicines using the Fisher’s exact test and the Pearson chi-square test at a 5% significance level. We calculated proportions of PLHIV with controlled blood pressure in each treatment arm at each month of follow up and estimated the closest time to BP control using the Kaplan Meier survival estimates considering the treatment protocol as one composite intervention.

We tested the proportional hazards assumption using scaled Schoenfeld residuals and it was met (*p* > 0.05). We therefore used Hazard ratios (HR) from the univariate and multivariable cox proportional hazards models to determine the predictors of time to first BP control at a 5% significance level. In our models, we included covariates known to influence blood pressure i.e., age, sex, BMI, smoking, immunological status (viral load suppression and CD4+ cell count) as well as ART regimens and duration on ART[19–21]

## Results

### Baseline characteristics

A total of 877 patients were enrolled in the study. Of these, 236 (30%) started treatment with Amlodipine 5 mg monotherapy and 641 (70%) started with Amlodipine 5 mg/Valsartan 80 mg duo-therapy. The mean age of study participants was 50.4 (SD = 9.5) and majority, 545 (62.1%) were female. There were no significant differences in sex and age between the two groups. Mean baseline systolic blood pressures for the monotherapy and duo-therapy groups were 145 (SD = 10.8) mmHg and 162 (SD = 17.7) mmHg respectively. Majority (69%) of the duo-therapy patients had baseline blood pressure between (160–179)/(100–109) with 22% and 8% having baseline blood pressure between (180–199)/(110–119) and (≥200)/(≥120) respectively. More than a third of the patients were overweight 339 (38.7%) followed by normal weight 283 (32.3%) and obese 224 (25.5%) with the minority being underweight 31 (3.5%). Of the enrolled patients, 396 (45.1%) had been on ART for <5 years, 395 (45.0%) between 5 and 10 years and 86 (9.8%) for more than 10 years. When evaluated for history of diagnosed diabetes mellitus, 177 (20.2%) reported having diabetes mellitus. There was no significant difference in frequency of smoking, ART combinations, most recent viral loads and baseline CD4 cell counts between the patients started on monotherapy versus duo-therapy (Table 1).

**Table 1:** Baseline demographic and clinical characteristics for patients enrolled in the study.

	Patients started on Amlo 5 (*N* = 236)	Patients started on Amlo5Val80 (*N* = 641)	Total (*N* = 877)	*P* value
Gender				
Female	151 (63.98)	394 (61.47)	545 (62.14)	0.496
Male	85 (36.02)	247 (38.53)	332 (37.86)	
Age in years, Mean (SD)	50 (9.67)	51 (9.47)	50.4 (9.52)	0.649
Age categories, Count (%)				
<30	0 (0.00)	4 (0.62)	4 (0.46)	0.681
30–39	28 (11.86)	67 (10.45)	95 (10.83)	
40–49	89 (37.71)	232 (36.19)	321 (36.60)	
50 and older	119 (50.42)	338 (52.73)	457 (52.11)	
Baseline BP in mm Hg, Mean (SD)				
Systolic	145 (10.82)	162 (17.74)	158 (17.78)	**<0.001**
Diastolic	92.71 (7.78)	102 (11.36)	99 (11.36)	**<0.001**
BP categories				
140–159/90–99	236 (100)			
160–179/100–109		443 (69.11)		
180–199/110–119		141 (22)		
≥200/≥120		57 (8.89)		
Baseline BMI, Count (%)				
Underweight (<19.0)	12 (5.08)	19 (2.96)	31 (3.53)	**0.020**
Normal weight (19.0 to <25.0)	76 (32.20)	207 (32.29)	283 (32.27)	
Overweight (25.0 to <30.0)	75 (31.78)	264 (41.19)	339 (38.65)	
Obese (>30.0)	73 (30.93)	151 (23.56)	224 (25.54)	
History of smoking, Count (%)				
Ever smoked	15 (6.36)	63 (9.83)	78 (8.89)	0.109
Baseline CD4 category, Count (%)				
<50	45 (19.07)	97 (15.13)	142 (16.19)	0.196
50 to <100	10 (4.24)	47 (7.33)	57 (6.50)	
100 to <200	55 (23.31)	165 (25.74)	220 (25.09)	
≥200	126 (53.39)	332 (51.79)	458 (52.22)	
Baseline ART regimen, Count (%)				
AZT-3TC-NVP	96 (40.68)	262 (40.87)	358 (40.82)	0.924
AZT-3TC-EFV	44 (18.64)	107 (16.69)	151 (17.22)	
TDF-3TC-NVP	19 (8.05)	62 (9.67)	81 (9.24)	
TDF-3TC-EFV	59 (25.00)	159 (24.80)	218 (24.86)	
Other	18 (7.63)	51 (7.96)	69 (7.87)	
Duration of ART, Count (%)				
<5years	90 (38.14)	306 (47.74)	396 (45.15)	**0.028**
5 to 10years	123 (52.12)	272 (42.43)	395 (45.04)	
>10years	23 (9.75)	63 (9.83)	86 (9.81)	
Viral load, copies/ml, Count (%)				
<1000	235 (99.58)	634 (98.91)	869 (99.09)	0.690
1000 and more	1 (0.42)	7 (1.09)	8 (0.91)	
History of DM, Count (%)				
Yes	36 (15.25)	141 (22.00)	177 (20.18)	**0.027**
Disclosure, Count (%)				
Yes	112 (47.46)	321 (50.08)	433 (49.37)	0.491

*BMI* Body mass index, *ART* Antiretroviral therapy, *TDF* Tenofovir, *3TC* Lamivudine, *DTG* Dolutegravir, *ABC* Abacavir, *EFV* Efavirenz, *NVP* Nevirapine, *DM* Diabetes mellitus.

Clinically significant differences in population characteristics have been Bolded.

### Blood pressure control at the different steps of the treatment protocol

Among the patients initiated on monotherapy (*N* = 236), 156 (66%) attained BP control within a month of treatment. Among the 80 patients with uncontrolled BP after the first month, 52 (22%) were controlled in the second month and 18 (8%) in the third month. Cumulatively, 66% of the patients were controlled in the first month, 88% by the second month and 96% by the third month. Only 7 (3%) patients failed to attain BP control after five months of follow up and 2(1%) did not complete follow up because they were transferred out to get care from another clinic (Table 2).

**Table 2:** Numbers and proportions of patients with controlled BP at the different protocol steps for the two groups over the follow up period.

Blood pressure control per month of treatment	Mono-therapy (*N* = 236)		Duo-therapy (*N* = 641)		Total
	Proportion controlled per treatment step	Cumulative BP control per treatment step	Proportion controlled per treatment step	Cumulative BP control per treatment step	Cumulative BP control per treatment step for the whole cohort
Achieved BP control in step 1	156 (66%)	66%			
Achieved BP control in step 2	52 (22%)	88%	362 (56%)	56%	518 (59%)- Controlled in month 1
Achieved BP control in step 3	18 (8%)	96%	173 (27%)	83%	743 (85%)- Controlled in month 2
Achieved BP control in step 4	-	96%	34 (5%)	88%	795 (91%)- Controlled in month 3
Achieved BP control in step 5	1 (0%)	97%	15 (2%)	90%	811 (92%)- Controlled in month 4
Not controlled after 5^th^ month	7 (3%)		40 (7%)		
Study drop outs before BP control	2 (1%)^a^		17 (3%)^b^		

^a^2 patients transferred out.

^b^2 patients lost to follow up, 10 patients transferred out, 1 defaulted treatment due to side effects (persistent lower limb swelling).

For the patients started on duo-therapy (*N* = 641), 362 (56%) attained BP control within the first month. Among the 279 patients who failed to attain BP control after the first month, 173(27%) attained BP control after the second month, 34 (5%) in the third month and 15 (2%) in the fourth month. Cumulatively 56% patients achieved blood pressure control in the first month, 83% by the second month, 88% by the third month and 90% by the fourth month. After four months of follow up, 40 (7%) patients had not yet attained BP control and 17 (3%) did not complete the follow up duration (2 patients lost to follow up, 4 patients died, 10 patients transferred out, 1 stopped medication due to persistent lower limb edema) (Table 2).

### Overall, probability of blood pressure control per month of follow up among all enrolled patients considering the treatment protocol as one composite intervention

**Fig. 3:**
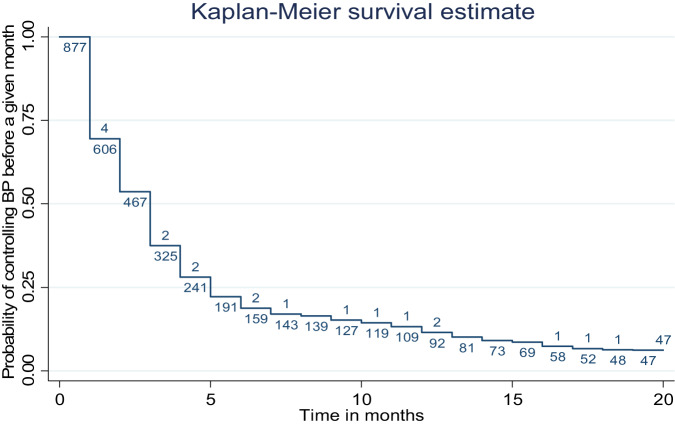
Kaplan Meier survival curve for time to first BP control among participants considering the treatment protocol as one composite. The figure shows cumulative probability of blood pressure control over time with the adapted hypertension treatment protocol. BP blood pressure.

The probability of blood pressure control was 30%, 50%, 70%, 80% and 85% at one, two, three, four and five months of follow up respectively (Fig. 3).

### Socio-demographic and clinical predictors to time to blood pressure control evaluating the tested treatment protocol as one composite intervention

We determined a high mean baseline systolic blood pressure (adjusted hazard ratio (aHR) 0.995, 95% Confidence Interval (95% CI): 0.989, 0.999, *P* value (p): 0.045), having a baseline ART regimen as tenofovir/ lamivudine/efavirenz (aHR: 0.764, 95% CI: 0.637, 0.917, *p* = 0.004) was associated with longer time to blood pressure control. Being on ART for more than 10 years was associated with a shorter time to blood pressure control (aHR: 1.456 95% CI; 1.126, 1.883) (Table 3).

**Table 3:** Predictors of time to first BP control.

Characteristic	Unadjusted HR [CI]	*P* value	Adjusted HR [CI]	*P* value
Gender				
Female	Ref		Ref	
Male	0.953 [0.827, 1.099]	0.516	0.969 [0.831, 1.129]	0.685
Age categories, Count (%)				
18–39	Ref		Ref	
40–49	1.017 [0.803, 1.289]	0.883	0.929 [0.728, 1.186]	0.557
50 and older	1.079 [0.859, 1.354]	0.510	0.926 [0.726, 1.182]	0.537
Baseline BP in mm Hg, Mean (SD)				
Systolic	0.991 [0.986, 0.994]	**0.000**	0.995 [0.989, 0.999]	**0.045**
Diastolic	0.985 [0.979,0.991]	**0.000**	0.994 [0.986, 1.001]	0.137
HTN treatment step started				
Started on step 1	Ref		Ref	
Started on step 2	0.861 [0.738, 1.004]	0.057	1.044 [0.872, 1.250]	0.637
Baseline BMI, Count (%)				
Underweight (<19.0)	Ref		Ref	
Normal weight (19.0 to <25.0)	1.003 [0.679, 1.481]	0.985	1.126 [0.758, 1.672]	0.556
Overweight (25.0 to <30.0)	1.031 [0.701, 1.518]	0.875	1.154 [0.779, 1.710]	0.474
Obese (>30.0)	0.893 [0.601, 1.326]	0.577	0.999 [0.668, 1.496]	0.999
History of smoking, Count (%)				
Never smoked	Ref		Ref	
Ever smoked	1.045 [0.821, 1.329]	0.718	1.011 [0.784, 1.131]	0.933
Baseline CD4 category, Count (%)				
<50	Ref		Ref	
50 to <100	1.037 [0.752, 1.430]	0.823	1.066 [0.766, 1.482]	0.704
100 to <200	1.060 [0.852, 1.319]	0.600	1.010 [0.807, 1.264]	0.927
≥200	1.001 [0.823, 1.218]	0.986	1.071 [0.876, 1.307]	0.503
Baseline ART regimen, Count (%)				
AZT-3TC-NVP	Ref		Ref	
AZT-3TC-EFV	0.928 [0.762, 1.130]	0.461	0.956 [0.779, 1.172]	0.667
TDF-3TC-NVP	1.119 [0.872, 1.436]	0.374	1.194 [0.926, 1.539]	0.171
TDF-3TC-EFV	0.803 [0.674, 0.957]	**0.015**	0.764 [0.637, 0.917]	**0.004**
Other	0.675 [0.511, 0.890]	**0.005**	0.774 [0.583, 1.027]	0.076
Duration of ART, Count (%)				
<5years	Ref		Ref	
5 to 10years	1.340 [1.043, 1.722]	**0.022**	1.168 [0.904, 1.509]	0.233
>10years	1.741 [1.354, 2.238]	**0.000**	1.456 [1.126, 1.883]	**0.004**
Viral load, copies/ml, Count (%)				
Non-suppressed	Ref		Ref	
Suppressed	1.055 [0.472, 2.356]	0.896	0.666 [0.292, 1.515]	0.333
History of DM, Count (%)				
No	Ref		Ref	
Yes	0.748 [0.626, 0.893]	**0.001**	0.931 [0.776, 1.117]	0.442
Disclosure, Count (%)				
No	Ref		Ref	
Yes	0.891 [0.777, 1.023]	0.103	0.981 [0.851, 1.130]	0.788

aHR < 1 means Increased “risk of BP control”, therefore reduced time to BP control.

aHR > 1; Decreased “risk of BP control”, therefore Increased time to BP control.

Bolded *P* values show baseline characteristics that were significantly different between the two study groups.

## Discussion

In resource limited settings where fixed dose combination antihypertensive drugs, trained human resource as well as availability of facilities for blood pressure monitoring are scarce, adherence to universally agreed upon guidelines in the treatment of hypertension is challenging [9, 22, 23]. Because of the increasing burden of non-communicable diseases (NCDs) worldwide, the WHO developed a HEARTS technical package aimed at providing country ministries of Health with a strategic approach in improving cardiovascular disease (CVD) management in primary care settings [14]. We adopted this model and tailored it to our study clinic setting in Uganda evaluating its effectiveness by determining time to blood pressure control in patients started on monotherapy and those on duo-therapy [15].

More than half of the patients in each treatment group attained blood pressure control four weeks after starting treatment, with approximately 90% achieving BP control by the third month, irrespective of the anti-hypertensive regimen they received. Time to blood pressure control has been evaluated in different world settings comparing the efficacy of monotherapy versus duo-therapy anti-hypertensive treatment. In a large retrospective database cohort in the USA, up to about 55% of patients on monotherapy and 60% of those on fixed dose combination drugs were controlled by 400 days [24]. In another Malaysian cohort largely on monotherapy, BP control was achieved in 47% and 65% at 6 and 12 months respectively [25]. Our protocol was implemented in a real world setting with integrated adherence support for both ART and hypertension treatment. This ongoing routine adherence counseling could have influenced the high rates of BP control observed in our study as compared to the rates in the above cohorts.

Hypertension is one of the most attributable traditional risk factors responsible for atherosclerotic CVD in person living with HIV [26]. Our study population exhibited a high prevalence of CVD risk factors underlining the need for more effective blood pressure control. A large percentage of the patients were overweight and obese with a mean age of 50 years. Additionally, majority had been exposed to ART for more than five years. Long term exposure to ART including nucleoside reverse transcriptase inhibitors (NRTIs) and protease inhibitors (PIs) has also been linked to heightened CVD risk [27, 28].

Multiple studies have evaluated the contribution of conventional risk factors to hypertension in PLHIV and determined that most factors cut across both HIV infected and uninfected populations. Additionally, multiple studies have found an increased risk of hypertension with longevity on anti-retroviral therapy [29–32]. However, the association of these factors to BP control is largely understudied in PLHIV [19, 33, 34]. In our study, we determined that patients who had been on ART for ≥10 years tended to have a shorter time to blood pressure control. This could be attributed to transferred adherence to anti-hypertensive therapy following years of ART adherence counseling.

We had some limitations in execution of this study. This was an intervention in a single HIV clinic site and hence the results may not be generalizable to the general HIV positive population. This is because hypertension in different regions of sub-Saharan Africa may be driven by different factors such as diet, physical activity as well as environment. Additionally, during the time of intervention, availability of hypertension drugs as well as emphasis on patient education on lifestyle modification was ensured as part of the WHO HEARTS model that was being tested which may lead to overestimation of the success of the rates of BP control as compared to other public health care settings in Africa with frequent drug stockouts as well as limited personnel. Despite the limitations, we demonstrated that this simplified protocol was effective in attaining timely BP control and minimizing pill burden in a pragmatic resource limited setting.

## Conclusions

In this study, an adapted WHO HEART treatment protocol attained timely BP control among hypertensive PLHIV. Additionally, we demonstrated that on initiation of Amlodipine 5mg-monotherapy in PLHIV with stage I hypertension and Amlodipine 5 mg/Valsartan 40 mg duo-therapy in patients with ≥ stage 2 hypertension, there was reasonably high rates of blood pressure control in the first month. The use of monotherapy anti-hypertensive treatment can be a viable undertaking in similar clinical settings to limit treatment cost and pill burden. Larger multi-center studies should be conducted to increase generalizability of the results.

## Summary

### What is known about the topic

- Persons living with HIV have a high prevalence of hypertension.
- Controlled hypertension reduces cardiovascular disease mortality and morbidity.
- Integrated management of HIV and hypertension improves hypertension control and sustains HIV control among hypertensive persons living with HIV.

### What this study adds

- The WHO HEARTS strategy was effective at achieving timely BP control among Persons living with HIV.
- The monotherapy anti-hypertensive treatment for stage I hypertension is a viable option to achieve BP control and limit pill burden in resource limited HIV care settings.

## Data Availability

The datasets used and/or analysed during the current study are available from the corresponding author on reasonable request.
